# Characterization of Mitochondrial DNA Methylation of Alzheimer’s Disease in Plasma Cell-Free DNA

**DOI:** 10.3390/diagnostics13142351

**Published:** 2023-07-12

**Authors:** Binrong Ding, Xuewei Zhang, Zhengqing Wan, Feng Tian, Jie Ling, Jieqiong Tan, Xiaoqing Peng

**Affiliations:** 1 Department of Geriatrics, The Third Xiangya Hospital, Central South University, Changsha 410000, China; dingbinrong@163.com; 2 Health Management Center, Xiangya Hospital, Central South University, Changsha 410000, China; 3 Hengyang Medical School, University of South China, Hengyang 421001, China; 4 The 8 Ward, The Ninth Hospital of Changsha, Changsha 410000, China; 5 Medical Functional Experiment Center, School of Basic Medicine, Central South University, Changsha 410000, China; lingjie@sklmg.edu.cn; 6 Center for Medical Genetics & Hunan Key Laboratory of Medical Genetics, School of Life Sciences, Central South University, Changsha 410000, China; 7 Institute of Molecular Precision Medicine, Xiangya Hospital, Central South University, Changsha 410000, China; 8 Hunan Key Laboratory of Molecular Precision Medicine, Changsha 410000, China; 9 Hunan International Scientific and Technological Cooperation Base of Neurodegenerative and Neurogenetic Diseases, Changsha 410000, China

**Keywords:** Alzheimer’s disease, mitochondrial DNA methylation, cell-free DNA, noninvasive diagnosis

## Abstract

Noninvasive diagnosis of Alzheimer’s disease (AD) is important for patients. Significant differences in the methylation of mitochondrial DNA (mtDNA) were found in AD brain tissue. Cell-free DNA (cfDNA) is a noninvasive and economical diagnostic tool. We aimed to characterize mtDNA methylation alterations in the plasma cfDNA of 31 AD patients and 26 age- and sex-matched cognitively normal control subjects. We found that the mtDNA methylation patterns differed between AD patients and control subjects. The mtDNA was predominantly hypomethylated in the plasma cfDNA of AD patients. The hypomethylation sites or regions were mainly located in mt-rRNA, mt-tRNA, and D-Loop regions. The hypomethylation of the D-Loop region in plasma cfDNA of AD patients was consistent with that in previous studies. This study presents evidence that hypomethylation in the non-protein coding region of mtDNA may contribute to the pathogenesis of AD and potential application for the diagnosis of AD.

## 1. Introduction

Alzheimer’s disease (AD) is the most prevalent neurodegenerative disease, characterized by cognitive impairment and personality changes that eventually lead to severe disability [1]. Its main pathological features comprise accumulation of extracellular Aβ plaques, the formation of intracellular neurofibrillary tangles, and the loss of neurons in the brain. Since these pathological features alone are insufficient to fully explain the onset and progression of the disease, biomarkers solely based on these characteristic pathological features may potentially lead to misdiagnosis or delayed diagnosis. Thus, the identification of new biomarkers for AD holds promise for improving current diagnosis of AD and ultimately improving the treatment and management of this devastating disease.

Mitochondria are essential organelles in cells that play an important role in pathogenesis of AD. These semi-autonomous organelles possess their own genome. Human mitochondrial DNA (mtDNA) is a double-stranded circular molecule comprising 16,569 bp and consists of both coding and non-coding regions. The coding region contains 37 genes that encode for 2 ribosomal RNAs (rRNAs), 22 transfer RNAs (tRNAs), and 13 proteins that are essential components of the respiratory chain. The non-coding region, known as the displacement loop or D-Loop, contains the mtDNA replication initiation site and transcription start site [2]. Various factors such as mtDNA mutations and deletions, abnormal morphology, disruption of calcium homeostasis, abnormal mitophagy, and impaired biogenesis can cause mitochondrial dysfunction, which can promote development of AD [3,4,5]. The mitochondrial cascade hypothesis of AD [6] proposes that genetic changes in mitochondrial genes resulting in impaired mitochondrial function are considered a primary mechanism of AD pathogenesis. Furthermore, previous studies have suggested that the innate immune system has a preponderant role in AD [7]. Innate immunity can be triggered by the non-methylated CpG sites of mtDNA [8,9]. Recent research has shown that epigenetic modifications of mitochondrial genes, which do not involve changes in the genetic sequence, may play a role in the development of neurodegenerative diseases [10]. However, it remains unknown whether alterations in mtDNA methylation can be observed in AD patients, and the correlation between mitochondrial DNA methylation changes and AD remains unclear. Therefore, analyzing the methylation features of mtDNA can provide valuable insights for a better understanding of the pathogenesis of AD.

Similar to the nuclear genome, the mitochondrial genome may regulate its function through various epigenetic modifications. As a result, there is a growing interest in investigating the role of mtDNA epigenetic modifications in the pathogenesis of AD [4,11,12,13]. Blanch et al. [14] found an increased methylation level in the D-Loop region of the entorhinal cortex in AD patients compared to control subjects. Stoccoro et al. [15,16] found a decreased methylation level in the D-Loop region of peripheral blood in AD patients compared to control subjects. These findings indicate that methylation changes in the D-Loop region can be detected in both brain tissue and peripheral blood of AD patients, suggesting that peripheral blood methylation may serve as an accessible surrogate for certain brain-specific methylation changes. Furthermore, Devall et al. [17] demonstrated a correlation in mtDNA methylation patterns between the cortex, cerebellum, and blood. Therefore, analyzing the epigenetic characteristics of mtDNA methylation profile in peripheral blood has the potential to offer a noninvasive approach for the early detection and monitoring of AD.

Circulating cell-free DNA (cfDNA) refers to extracellular DNA that can be found in various bodily fluids and secretions, including blood, urine, and saliva [18,19]. It can be released into the peripheral blood by various tissues and organs, such as the brain, liver, and lungs, during tissue and cell apoptosis and necrosis. By identifying the characteristics of cfDNA derived from different tissues and organs in peripheral blood, it becomes possible to trace and evaluate pathological changes in the corresponding tissues [20,21,22,23]. Therefore, we speculate that analyzing the epigenetic characteristics of mtDNA methylation profile in plasma cfDNA between AD patients and control subjects, which contains DNA fragments from various tissues and organs, holds greater potential for identifying mitochondria methylation changes linked to AD. It may potentially provide biomarkers that reflect the presence of AD, thereby facilitating accurate diagnosis and personalized treatment strategies.

In this study, we hypothesized that by comparing the differences in mtDNA methylation patterns between AD patients and control subjects in plasma cfDNA samples, it is possible to obtain mtDNA methylation features associated with AD, thereby providing candidate biomarkers for noninvasive diagnosis of AD. To accomplish this, we utilized whole-genome bisulfite sequencing (WGBS) to extract mtDNA methylation profiles from plasma cfDNA samples obtained from both AD patients and cognitively normal control subjects. Subsequently, we performed a differential analysis of mtDNA methylation levels between these two groups to characterize the mtDNA methylation alterations present in plasma cfDNA.

## 2. Materials and Methods

### 2.1. Participants and Blood Sample Collection

In the current study, 57 individuals, comprising 31 AD patients and 26 age- and sex-matched controls (Table 1 and Table S1), were enrolled. All AD patients were recruited at the Ninth Hospital of Changsha and fulfilled the diagnostic criteria for “probable AD” as defined by the NINCDS-ADRDA (National Institute of Neurological and Communicative Diseases and Stroke/Alzheimer’s Disease and Related Disorders Association) [24]. Volunteer subjects with normal cognition, matched for age and sex to the AD patients, were recruited as control subjects. Exclusion criteria for the study included the following: (1) individuals under the age of 60 years; (2) individuals with a history of diagnosis other than AD (e.g., frontotemporal dementia, vascular dementia, Parkinson’s disease, Huntington’s disease, dementia with Lewy bodies, or hippocampal sclerosis); (3) individuals with cognitive impairment attributable to psychosis (e.g., depression), drug abuse, alcohol use, medication use, or medical illness; (4) individuals who were unable to complete the cognitive assessment due to a medical illness.

Informed and written consent was obtained from each subject before inclusion in the study that was approved by the Ethics Committee of Xiangya Hospital of Central South University (Approval No: 201706826).

### 2.2. Plasma cfDNA Extraction and Quantification

A total of 10 mL of venous blood sample was collected from each subject in a Streck cfDNA blood collection tube (Catalog No. 218997, Streck, La Vista, NE, USA), which is designed to stabilize and preserve cfDNA in the blood. The plasma was then extracted from the blood by centrifuging at 1600× *g* for 10 min at 4 °C. The resulting supernatant was carefully transferred into a 1.5 mL Eppendorf micro-centrifuge tube without disturbing the buffy coat and then underwent a second centrifugation at 16,000× *g* for 10 min at 4 °C to remove any remaining cell debris. The cfDNA was subsequently isolated from the plasma using the QIAGEN circulating nucleic acid extraction kit (Catalog No. 55114, QIAGEN, Valencia, CA, USA) and quantified using a Qubit 3.0 Fluorometer.

### 2.3. DNA Methylation Library Preparation

The extracted cfDNA was subjected to bisulfite conversion using the EZ DNA Methylation-Lightning Kit (Catalog No. D5030, Zymo Research, Irvine, CA, USA) per the manufacturer’s instructions. Following bisulfite conversion, a post-bisulfite whole genome bisulfite sequencing (WGBS) library was constructed for each sample using the NEBNext Ultra II DNA Library Prep Kit for Illumina (Catalog No. E7645, NEB, Ipswich, MA, USA) and NEBNext Multiplex Oligos for Illumina (Catalog No. E7535, NEB, USA) per the manufacturer’s instructions. The resulting libraries were quantified using a Qubit 3.0 Fluorometer.

### 2.4. Sequencing and Data Processing

Libraries were sequenced on an Illumina Nova6000 (Illumina Inc., New York, NY, USA) using the PE150 sequencing strategy. After sequencing, the quality assessment of the obtained reads was carried out by FastQC (v0.11.5). To improve data quality, low-quality bases and adapter sequences were removed using Trim Galore (v0.6.5). Subsequently, the trimmed reads were mapped to the hg19 reference genome with Bismark (v0.19.0). Following alignment, de-duplication was performed to eliminate PCR duplicates resulting from library preparation. To determine the DNA methylation status at each CpG site in the genome, methylation calling was performed using the aligned and processed reads. For each CpG site, the depth was calculated based on the reads mapped to both strands, and methylation level was calculated as the ratio of methylated reads to the sum of methylated and unmethylated reads. This ratio provided an estimate of the methylation status at the specific CpG site, with values ranging from 0 (completely unmethylated) to 1 (completely methylated).

### 2.5. Statistical Analysis

The data analysis focused on the methylation alteration of mitochondrial DNA only. Depth of specific CpGs was obtained by merging reads mapped to both strands, and only CpG sites with read depth ≥ 5 were retained in the analysis. Differentially methylated CpG sites (DMCs) and differentially methylated regions (DMRs) were identified by generalized linear model (GLM) in a comparison between AD patients and control subjects. DMRs were genomic regions with differential methylation (*p*-value < 0.05) within 100 bp non-overlapping windows. Only CpG sites or methylated regions that were detected in at least five AD patients and five control subjects were included in the analysis. Furthermore, the mtDNA methylation alterations that were significantly different between AD patients and control subjects were analyzed based on gender using GLM. The relationship between mtDNA methylation levels and age was assessed using Spearman’s rank correlation. Differences in age were compared using Student’s *t*-test. Fisher’s exact test was performed to test differences in AD status and gender, as well as to evaluate the enrichment of trends identified within our data. Statistical significance was shown as * *p* < 0.05, ** *p* < 0.01. The Bonferroni method was used to adjust for multiple comparisons. Statistical Package for the Social Sciences (SPSS, Chicago, IL, USA) 25.0, R-statistical tools version 4.0.3, and Prism software 8.0 were used for statistical analysis and visualization.

## 3. Results

### 3.1. MtDNA Methylation of AD Patients with Different Patterns Compared to Control Subjects

**Figure 1:**
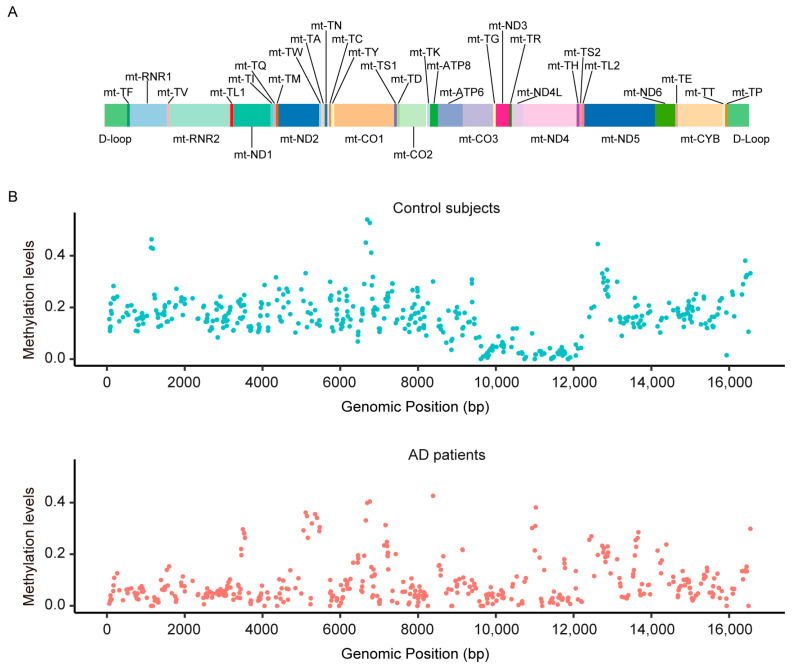
MtDNA methylation pattern of AD patients with different patterns compared to control subjects. (**A**) The genetic map of the human mitochondrial genome. (**B**) Scatter plot representation of mtDNA methylation levels of each CpG site in the control group (*n* = 26) and AD groups (*n* = 31).

We performed mitochondrial DNA (mtDNA) methylation analysis on the plasma cfDNAs of 31 AD patients and 26 age- and sex-matched cognitively normal control subjects (Table 1). A total of 406 CpG sites, detected in at least 5 AD patients and 5 control subjects, were included in further analyses. In general, the mtDNA methylation pattern appeared to be different in a comparison between AD patients and control subjects. The ranges of methylation level in the CpG sites were 0~0.426 in AD patients and 0~0.540 in control subjects (Figure 1). The average methylation level of 406 sites was lower in AD patients (mean = 0.127 standard deviation [SD] = 0.161), compared with control subjects (mean = 0.138, SD = 0.151).

Further, we investigated the variability of each CpG site in mtDNA levels by calculating the coefficient of variation (CV), which is defined as the ratio of SD to the mean. This was determined for each CpG site separately in AD patients and control subjects, following the study conducted by Devall et al. [25]. Of the 406 CpG sites we assessed, 383 had CVs greater than 1 in AD patients and 321 were greater than 1 in control subjects. In both AD patients and control subjects, more than 79.0% of the assessed CpG sites showed inter-individual variation, suggesting a widespread distribution of mtDNA methylation patterns among individuals. Moreover, the variations were found to be greater in AD patients compared to the control subjects (enrichment = 4.402, *p* = 1.01 × 10^−10^).

### 3.2. DMCs Were Predominantly Hypomethylated in Mt-CYB, Mt-RNR1, and Mt-RNR2 in Plasma CfDNA of AD Patients

**Figure 2:**
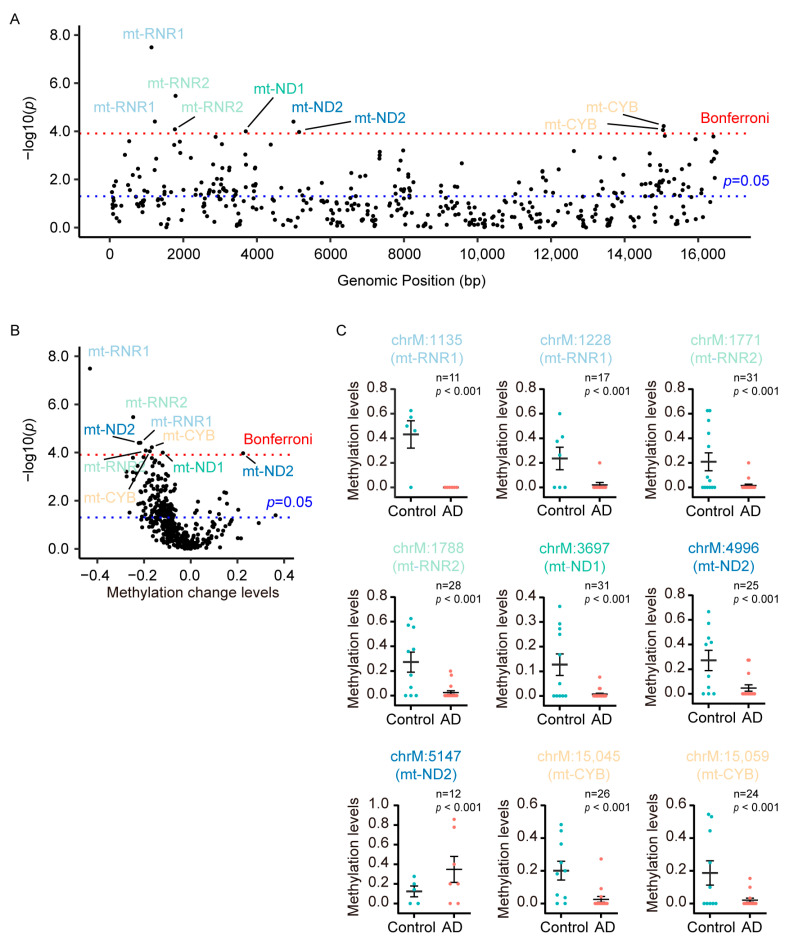
DMCs were predominantly hypermethylated in mt-CYB, mt-RNR1, and mt-RNR2 in plasma cfDNA of AD patients. (**A**) The scatter plot shows the −log *p* values obtained from the analysis of a GML performed on each CpG site in the mitochondrial genome, comparing AD patients (*n* = 31) and control subjects (*n* = 26). Red dashed line denotes the Bonferroni significance, while blue dashed line denotes *p* < 0.05 in the lower panel. (**B**) Volcano plots of methylation changes plotted against −log *p* value differences between AD patients (*n* = 31) and control subjects (*n* = 26). Red dashed line denotes the Bonferroni significance, while blue dashed line denotes *p* < 0.05. (**C**) The methylation level of the nine DMCs that passed the Bonferroni correction.

To investigate whether mtDNA methylation correlated with AD, we identified DMCs in the whole mitochondrial genome between AD patients and control subjects. In total, we identified 158 significant DMCs (*p* < 0.05) between AD patients and control subjects, of which 9 passed the Bonferroni correction test (Figure 2A). Interestingly, among these 158 significant DMCs, which mainly covered D-Loop, mt-ND1, mt-ND2, mt-ND4, mt-ND5, mt-CO1, mt-CYB, mt-RNR1, and mt-RNR2 regions, 146 DMCs (92.4%) were found to be hypomethylated in AD (Figure 2B). Nine DMCs that passed the Bonferroni correction (Bonferroni-adjusted *p*-value = 1.23 × 10^−4^) were mainly located in mt-RNR1, mt-RNR2, mt-ND1, mt-ND2, and mt-CYB. Among these DMCs, eight out of nine (88.9%) were found to be hypomethylated, while only one hypermethylated CpG site was identified in mt-ND2 in the plasma cfDNA of AD patients (Figure 2C).

Moreover, to examine age and gender effect on the methylation levels of the nine DMCs that passed the Bonferroni correction, analysis of the difference in methylation levels between male subjects and female subjects was performed by generalized linear model (GLM). Furthermore, Spearman’s rank correlation was utilized to explore the relationship between the methylation levels of these DMCs and age. In the total sample, the methylation levels of these nine DMCs were higher in males compared to those in females, but only three of them were statistically significant (Table 2). Sample stratification into AD patients and control subjects showed that the significant differences in methylation levels were predominantly observed in control subjects (Table 2). Regarding the association between age and methylation levels, a positive correlation was observed for one DMC (chrM: 15,059) in the total sample (Table 3). But this correlation had no significant correlation between methylation levels and age in both AD patients and control subjects (Table 3).

### 3.3. DMRs Were Predominantly Hypomethylated in D-Loop, Mt-RNR2, Mt-TT/Mt-TP in Plasma CfDNA of AD Patients

**Figure 3:**
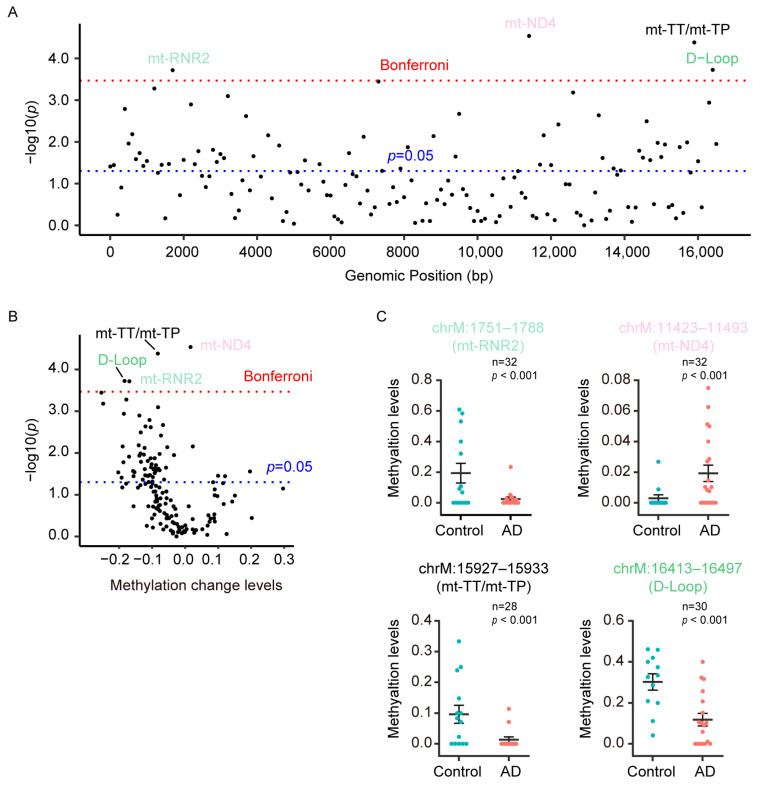
DMRs were predominantly hypomethylated in D-Loop, mt-RNR2, mt-TT/mt-TP in plasma cfDNA of AD patients. (**A**) The scatter plot shows the −log *p* values obtained from the analysis of a GML performed on each DMR in the mitochondrial genome, comparing AD patients (*n* = 31) and control subjects (*n* = 26). Red dashed line denotes the Bonferroni significance, while blue dashed line denotes *p* < 0.05 in the lower panel. (**B**) Volcano plots of methylation changes plotted against −log *p* value differences between AD patients (*n* = 31) and control subjects (*n* = 26). Red dashed line denotes the Bonferroni significance, while blue dashed line denotes *p* < 0.05. (**C**) The methylation level of the four DMRs that passed the Bonferroni correction.

Since DMRs are regions that contain multiple methylation sites and are thought to be more powerful for transcriptional regulation, we also identified DMRs in the mitochondrial genome in a comparison between AD patients and control subjects. In this analysis, we identified 58 significant DMRs (*p* < 0.05), of which four passed the Bonferroni correction (Figure 3A). Among these 58 significant DMRs, 53 DMRs (91.4%) were found to be hypomethylated in AD patients (Figure 3B). The four DMRs that passed the Bonferroni correction (Bonferroni-adjusted *p*-value = 3.40 × 10^−4^) were located in D-Loop, mt-ND4, mt-RNR2, and mt-TT/mt-TP, and three of them displayed hypomethylation in the plasma cfDNA of AD patients (Figure 3C).

To investigate the influence of age and gender on the methylation levels of the DMRs that passed the Bonferroni correction, we utilized the GLM to analyze the differences in methylation levels between male subjects and female subjects. Additionally, Spearman’s rank correlation was used to investigate the association between the methylation levels of these DMRs and age. In the total sample, the methylation levels of these four DMRs were higher in male subjects compared to those in female subjects, but only one (chrM: 15,927–15,933) of them exhibited statistically significant differences (Table 4). Sample stratification into AD patients and control subjects revealed that the significant differences in methylation levels were observed in control subjects (Table 4). In total sample, the methylation levels of chrM: 11,423–11,493 had a significant positive correlation with age, while the methylation levels of chrM: 15,927–15,933 and chrM: 16,413–16,497 had significant negative correlations with age (Table 5). However, there were no significant correlation between methylation levels and age in both AD patients and control subjects (Table 5). The methylation levels in chrM: 11,423–11,493 were significantly negatively correlated with age in AD patients, but this was different to the correlation found in the total sample.

### 3.4. The Methylation Level Decreased in the Mt-rRNA, Mt-tRNA, and D-Loop Gene Regions in Plasma CfDNA of AD Patients

**Figure 4:**
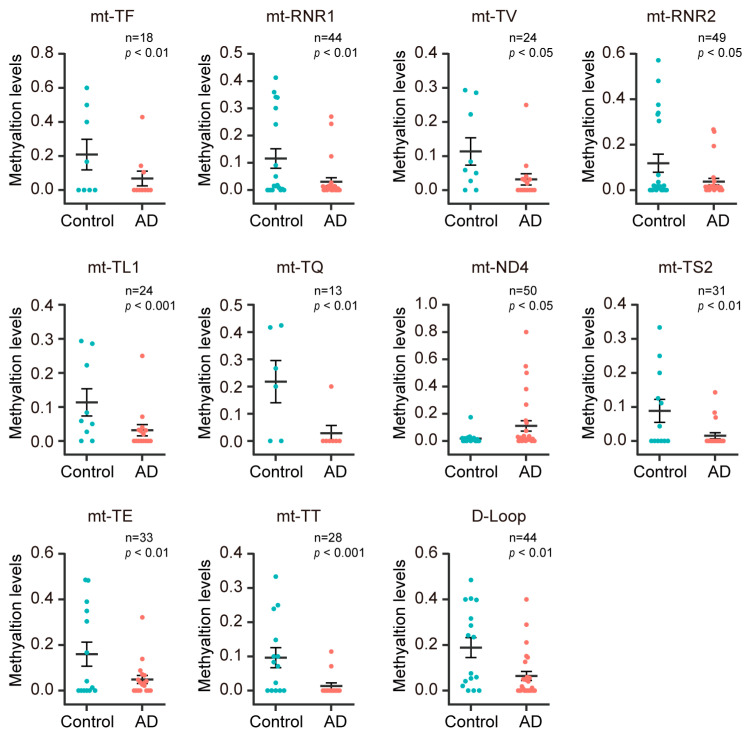
The methylation level decreased in the mt-rRNA, mt-tRNA, and D-Loop gene regions in plasma cfDNA of AD patients. The methylation levels in AD patients and control subjects for mt-TF, mt-RNR1, mt-TV, mt-RNR2, mt-TL1, mt-TQ, mt-ND4, mt-TS2, mt-TE, mt-TT, and D-Loop regions are shown.

To investigate the methylation changes of mitochondrial genomic regions in plasma cfDNA of AD patients, we conducted differential methylation analysis on mitochondrial genomic regions in a comparison between AD patients and the control subjects using GML. In 37 coding regions and 1 control region (D-Loop region) of the mitochondrial genome, 10 gene regions were excluded due to insufficient sample size. Among the remaining 28 gene regions, significant differences in methylation levels were observed between AD patients and the control subjects in 11 gene regions. Specifically, 10 gene regions (mt-TF, mt-RNR1, mt-TV, mt-RNR2, mt-TL1, mt-TQ, mt-TS2, mt-TE, mt-TT, and D-Loop) were found to be hypomethylated in AD patients, while 1 gene region (mt-ND4) showed hypermethylation in AD patients (Figure 4).

In order to explore the impact of age and sex on the methylation levels of eleven mitochondrial genomic regions that exhibited significant differences between AD patients and control subjects, GLM was performed to assess the differences in methylation levels between male and female subjects for these genomic regions. Furthermore, Spearman’s rank correlation was conducted to examine the relationship between the methylation levels of these genomic regions and age. In the total sample, male subjects displayed hypermethylation in three out of these eleven genomic regions, whereas female subjects exhibited hypermethylation in one genomic region (Table 6). Upon stratifying the sample into AD patients and control subjects, we observed that methylation alteration in mt-TF was driven by AD patients, while the methylation alteration in mt-TL1 and mt-TT were driven by control subjects (Table 6). Regarding the correlation analysis, we found a positive correlation between mt-ND4 methylation levels and age, and negative correlation between methylation levels in four genomic regions (mt-RNR1, mt-TL1, mt-TS2, mt-TT, and D-Loop) and age in the total sample (Table 7). Upon stratifying the sample into AD patients and control subjects, we determined that the correlation between mt-RNR1 and age was driven by AD patients, whereas the correlation between mt-TL1 and age was driven by control subjects (Table 7).

**Figure 5:**
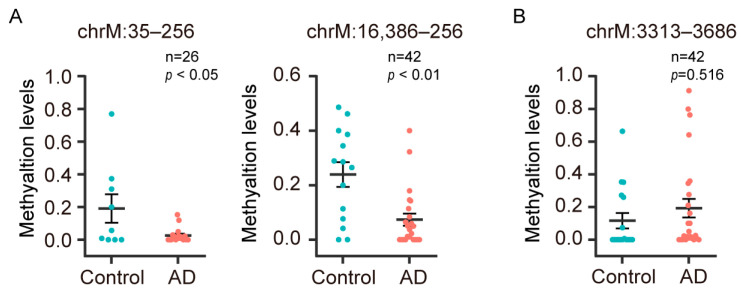
The methylation levels of D-Loop and mt-ND1 regions known to be associated with AD in AD patients and control subjects. The methylation levels in plasma cfDNA of AD patients and control subjects for D-Loop regions (**A**) and mt-ND1 regions (**B**) are shown.

By comparing the D-Loop and mt-ND1 regions, which are known to be associated with AD, we found that the hypomethylated region of D-Loop (chrM: 35–256) in peripheral blood DNA of AD patients [15,16] was also hypomethylated in the plasma cfDNA; the hypermethylated region of D-Loop (chrM: 16,386–256) in the entorhinal cortex of AD patients was hypomethylated in the plasma cfDNA (Figure 5A). We observed that the mt-ND1 region (chrM: 3313–3686), which is hypomethylated in the entorhinal cortex of AD patients [14], showed no difference in plasma cfDNA (Figure 5B). However, we identified hypomethylation in the DMCs and DMRs within the mt-ND1 from the plasma cfDNA of AD patients (Tables S2 and S3).

## 4. Discussion

In this study, we analyzed mtDNA methylation profiles in plasma cfDNA samples obtained from AD patients and cognitively normal subjects to characterize mtDNA methylation alterations in AD. The aim was to identify specific mtDNA methylation changes that may be associated with AD pathology and could potentially serve as biomarkers for the detection of AD. Unlike previous studies that focused on a limited number of gene regions or CpG sites within those gene regions, our study provides a comprehensive mapping of mtDNA methylation in AD patients. We found that the global methylation level of mtDNA was low in all subjects. MtDNA methylation patterns were widely distributed among individuals, with more than 79.0% of CpG sites exhibiting inter-individual variation. Notably, this variation was more common in AD patients. MtDNA hypomethylation was dominant in plasma cfDNA of AD patients compared with controls and was mainly located in non-protein coding regions of mitochondria. In the mitochondrial protein-coding region, hypermethylation of mt-ND4 was only observed in the plasma cfDNA of AD patients.

Age and gender are widely recognized as risk factors for AD [26]. Furthermore, age and gender have been found to potentially influence the mtDNA methylation levels in AD patients. Several studies have reported modifications in mtDNA methylation during senescence and aging [27]. Specifically, the D-Loop region was observed to be demethylated in senescent cells compared to proliferative endothelial cells [28]. D’Aquila et al. [29] observed a positive association between mt-RNR1 methylation levels in blood cells and age, particularly in elderly women, while Mawlood et al. [30] found a negative association between mt-RNR1 methylation levels in peripheral blood and age. Yu et al. [31] discovered hypomethylation in mt-RNR1 and mt-COX1 in senescent human heart mesenchymal stem cells (HMSCs). Sun et al. [32] found that MT-CO2 methylation levels increased with the senescence of HMSCs. Devall et al. [25] observed age- and gender-related patterns of mtDNA methylation in the superior temporal gyrus and cerebellum. MtDNA methylation tended to increase with age. Moreover, mtDNA hypomethylation was predominantly observed in females. In our study, we observed a predominant occurrence of mtDNA hypomethylation in the plasma cfDNA of AD patients. However, we found little correlation between age and sex for this hypomethylation alteration, suggesting that the observed methylation changes in mtDNA may primarily be associated with AD rather than being solely influenced by age and gender. These findings highlight the importance of considering mtDNA methylation as a potential target for further investigation in AD.

The existence of methylation in mammalian mtDNA remains a subject of debate. Previous studies utilizing bisulfite sequencing or pyrosequencing have reported relatively low levels of mtDNA methylation in the D-Loop region and the mt-RNR1 gene across different human cell and tissue types, with estimated levels ranging from 1% to 34% [25,33,34,35,36]. While a large number of studies have reported the presence of methylation in mtDNA, some studies have reported the absence of methylation in mtDNA [37,38]. In this study, methylation levels in all CpG sites ranged from 0% to 54%. When focusing on the methylation levels in the D-Loop region, the observed range was consistent with previous findings, ranging from 0% to 38%. Devall et al. [17] demonstrated that mtDNA methylation patterns are relatively low and conserved in the superior temporal gyrus and cerebellum. However, in our study, we found that mtDNA methylation patterns were consistently low but widely distributed among individuals. This variability can be attributed to differences in methylation patterns across various cell or tissue types. Plasma cfDNA contains DNA fragments from various cells and tissues, resulting in a relatively high diversity of methylation patterns for mtDNA in plasma cfDNA.

Due to the lack of studies on mitochondrial DNA methylation profiling in AD patients, we cannot compare our results with previous studies. However, compared to previous findings for the D-Loop and mt-ND1 regions, we found hypomethylation in the D-Loop region of plasma cfDNA from AD patients, which is in line with findings in peripheral blood of AD patients [15,16] and the hippocampus in an AD mouse model [39]. Although the methylation level of the mt-ND1 region has been reported to decrease in the entorhinal cortex of AD patients [14], we did not find a significant difference in plasma cfDNA between AD patients and control subjects. However, hypomethylation was observed in DMCs and DMRs within the mt-ND1 from the plasma cfDNA of AD patients, consistent with previous study [14]. Moreover, Stoccoro et al. [15] reported hypermethylation in the D-Loop region of blood cells in control subjects compared to AD patients at advanced stages of the disease, but not in those at early stages. Interestingly, Blanch et al. [14] found a dynamic pattern of D-Loop methylation pattern during the progression of AD pathology in an AD mouse model. They observed lower D-Loop methylation levels in the cerebral cortex of AD mice at 3 months old, higher levels at 6 months old, and lower levels again at 12 months old. The results from Stoccoro et al. [15] and Blanch et al. [14] suggested that D-Loop methylation levels altered during the progression of AD, and these alterations can be identifiable both in brain and in blood cells. Based on our study, we not only confirmed hypomethylation in the D-Loop region of plasma cfDNA from AD patients, providing additional support for the association between methylation alterations in this region and AD, but we also observed mtDNA methylation changes beyond the D-Loop region that were associated with AD, thereby expanding the current understanding of mtDNA methylation alterations in AD. These findings may suggest that mtDNA methylation alterations could potentially serve as promising epigenetic markers in AD. However, it is crucial to further explore how these mtDNA methylation changes are linked to the progression of the disease.

Increasing evidence suggests that changes in mtDNA methylation could regulate gene expression and mtDNA copy number [14,39,40]. For instance, Martorell et al. [41] found that patients with autism spectrum disorders accompanied by intellectual disability showed a significantly lower mtDNA copy number than control subjects in both mt-ND1 and mt-ND4 genes. Blanch et al. [14] investigated the entorhinal cortex of eight patients with AD, suggesting that hypomethylation of mt-ND1 could promote its expression. Xu et al. [39,40] found that increased methylation levels of mt-CYTB, mt-COX2, and 12S rRNA gene methylation levels in APP/PS1 transgenic mice may not only reduce its expression but also decreased the mtDNA copy number. Moreover, a study of mitochondrial RNA performed by Kim et al. [42] found that mt-RNAs, including MT-ND1~6 mRNAs and other protein-coding and mt-tRNAs, were significantly elevated in plasma extracellular vesicles of AD patients. This result supports the finding of our study, which showed a decrease in the methylation levels of mt-ND1, mt-ND2, mt-ND5, mt-ND6, and various mt-tRNAs in plasma cfDNA of AD patients. The proteins encoded by mt-ND1~6 genes are integral to the formation of mitochondrial complex I, which is the first enzyme of the mitochondrial electron transport chain. This complex is a major contributor to the generation of the proton gradient across the mitochondrial inner membrane, which drives ATP production. Importantly, this complex is closely associated with tau load [43]. Moreover, Trushina et al. [44,45] have demonstrated that inhibiting complex I with an inhibitor can effectively reduce levels of Aβ and phospho-tau, thereby preventing cognitive decline in animal models of familial AD. Therefore, we speculate that the hypomethylation of mtDNA in plasma cfDNA of AD patients may contribute to increased expression of mitochondrial genes and possibly contribute to cognitive impairment. Additionally, mt-tRNA plays a central role in mitochondrial protein synthesis and the maintenance of respiratory chain function [46,47]. Mutations in mt-tRNA genes have been reported to be associated with both AD and Parkinson’s disease [48,49]. The D-Loop is a non-coding region of mitochondria that plays a crucial role in regulation of mitochondrial genome replication and expression. Studies have shown that D-Loop methylation levels negatively correlate with gene expression [50,51] and mtDNA copy number [40,52,53,54,55,56]. Hypomethylation in the D-Loop region of plasma cfDNA from AD patients suggests elevated mRNA levels in mitochondrial genes, as confirmed by a mitochondrial RNA study performed by Kim et al. [42]. These data provide evidence for the potential involvement of mitochondrial methylation in the development of AD, and suggest that exploring mtDNA methylation alterations in plasma cfDNA as biomarkers for diagnosing AD is feasible and reliable. Furthermore, since the innate immune system has a preponderant role in AD, while the non-methylated CpG sites of mtDNA can trigger innate immune responses, the hypomethylation of mtDNA may represent a potential mechanism for the activation of innate immune responses, thereby potentially contributing to the onset of AD.

Although we identified differences between mtDNA methylation patterns in AD patients and control subjects, suggesting a potential role of mtDNA methylation alterations in the development of AD, there are several limitations in our study. The sample size for measuring mtDNA methylation was relatively small, and larger samples are required to validate the reliability of the results. Furthermore, given the distinct genetic pattern of nuclear insertions of mitochondrial origin (NUMT) in comparison to mtDNA [57], it is important to eliminate their interference while utilizing mtDNA for investigating the pathogenesis of AD in future studies. In the future work, it is crucial to determine whether mtDNA methylation is a primary event or a consequence of mitochondrial dysfunction in AD. In conclusion, our study expands the understanding of mtDNA methylation alterations in AD and their potential role in the pathogenesis of the disease. These findings shed new light on the search for biomarkers of AD from the perspective of mitochondrial methylation.

## Figures and Tables

**Table 1:** Demographic characteristics of the study population.

	Control Subjects (*n* = 26)	AD Patients (*n* = 31)	*p*-Value
Age (mean ± SD)	77.3 ± 4.8	80.3 ± 6.6	0.056 ^a^
Gender (F/M)	13/13	14/17	0.793 ^b^

^a^ Student’s *t*-test; ^b^ Fisher’s exact test. Data are presented as mean ± SD.

**Table 2:** Effect of gender on the methylation levels of DMCs that passed the Bonferroni correction.

		Total			Control			AD		
		*N*	Mean	*p* Value	*N*	Mean	*p* Value	*N*	Mean	*p* Value
chrM: 1135 (mt-RNR1)	Male	6	0.276	0.267	4	0.414	—	2	0.000	1.000
	Female	5	0.100		1	0.500		4	0.000	
chrM: 1228 (mt-RNR1)	Male	11	0.134	0.021	4	0.319	0.036	7	0.029	0.047
	Female	6	0.063		3	0.125		3	0.000	
chrM: 1771 (mt-RNR2)	Male	18	0.117	0.104	7	0.289	0.093	11	0.007	0.444
	Female	13	0.068		6	0.114		7	0.029	
chrM: 1788 (mt-RNR2)	Male	16	0.131	0.102	5	0.386	0.031	11	0.015	0.230
	Female	12	0.089		5	0.158		7	0.040	
chrM: 3697 (mt-ND1)	Male	17	0.072	0.163	7	0.176	0.034	10	0.000	0.102
	Female	14	0.022		4	0.042		10	0.014	
chrM: 4996 (mt-ND2)	Male	14	0.162	0.163	6	0.331	0.146	8	0.034	0.894
	Female	11	0.105		4	0.179		7	0.063	
chrM: 5147 (mt-ND2)	Male	7	0.379	0.005	4	0.155	—	3	0.678	0.010
	Female	5	0.080		1	0.000		4	0.100	
chrM: 15,045 (mt-CYB)	Male	14	0.122	0.566	7	0.225	0.266	7	0.019	0.762
	Female	12	0.059		3	0.145		9	0.030	
chrM: 15,059 (mt-CYB)	Male	13	0.144	0.006	6	0.295	0.000	7	0.014	0.911
	Female	11	0.026		4	0.025		7	0.027	

**Table 3:** Correlation between age and methylation levels of DMCs that passed the Bonferroni correction.

	Total			Control			AD		
	*N*	r	*p* Value	*N*	r	*p* Value	*N*	r	*p* Value
chrM: 1135 (mt-RNR2)	11	0.510	0.160	5	0.835	0.165	6	—	
chrM: 1228 (mt-RNR2)	17	0.349	0.203	7	0.399	0.433	10	0.675	0.046
chrM: 1771 (mt-RNR2)	31	0.234	0.222	13	0.430	0.163	18	−0.016	0.952
chrM: 1788 (mt-RNR2)	28	0.136	0.506	10	0.434	0.243	18	−0.004	0.988
chrM: 3697 (mt-ND1)	31	0.192	0.318	11	0.211	0.558	20	0.266	0.271
chrM: 4996 (mt-ND2)	25	0.299	0.166	10	0.346	0.362	15	0.354	0.214
chrM: 5147 (mt-ND2)	12	−0.591	0.072	5	−0.673	0.327	7	−0.480	0.335
chrM: 15,045 (mt-CYB)	26	0.285	0.177	10	0.405	0.279	16	0.286	0.301
chrM: 15,059 (mt-CYB)	24	0.439	0.041	10	0.567	0.111	14	0.525	0.066

**Table 4:** Effect of gender on the methylation levels of DMRs that passed the Bonferroni correction.

		Total			Control			AD		
		*N*	Mean	*p* Value	*N*	Mean	*p* Value	*N*	Mean	*p* Value
chrM: 1751–1788 (mt-RNR2)	Male	18	0.114	0.127	7	0.273	0.085	11	0.013	0.309
	Female	14	0.079		7	0.114		7	0.044	
chrM: 11,423–11,493 (mt-ND4)	Male	14	0.014	0.362	7	0.004	0.684	7	0.023	0.246
	Female	18	0.013		5	0.002		13	0.017	
chrM: 15,927–15,933 (mt-TT/mt-TP)	Male	14	0.076	0.005	6	0.166	0.002	8	0.009	0.909
	Female	14	0.034		8	0.044		6	0.019	
chrM: 16,413–16,497 (D-Loop)	Male	15	0.194	0.692	7	0.319	0.229	8	0.084	0.198
	Female	15	0.189		5	0.278		10	0.145	

**Table 5:** Correlation between age and methylation levels of DMRs that passed the Bonferroni correction.

	Total			Control			AD		
	*N*	r	*p* Value	*N*	r	*p* Value	*N*	r	*p* Value
chrM: 1751–1788 (mt-RNR2)	32	−0.359	0.051	14	0.287	0.342	18	0.172	0.508
chrM: 11,423–11,493 (mt-ND4)	32	0.559	0.001	12	−0.254	0.450	20	−0.553	0.014
chrM: 15,927–15,933 (mt-TT/mt-TP)	28	−0.556	0.003	14	0.139	0.650	14	0.429	0.143
chrM: 16,413–16,497 (D-Loop)	30	−0.584	0.001	12	0.555	0.076	18	−0.156	0.551

**Table 6:** Effect of gender on the methylation levels of mitochondrial genomic regions that showed significant difference between AD patients and the control subjects.

		Total			Control			AD		
		*N*	Mean	*p* Value	*N*	Mean	*p* Value	*N*	Mean	*p* Value
mt-TF	Male	10	0.203	0.003	5	0.300	0.062	5	0.107	0.002
	Female	8	0.039		3	0.056		5	0.029	
mt-RNR1	Male	22	0.082	0.447	10	0.136	0.539	12	0.036	0.600
	Female	22	0.053		9	0.093		13	0.026	
mt-TV	Male	11	0.133	0.115	5	0.253	0.050	6	0.033	0.811
	Female	13	0.060		5	0.058		8	0.061	
mt-RNR2	Male	24	0.093	0.352	11	0.151	0.390	13	0.043	0.710
	Female	25	0.056		11	0.086		14	0.032	
mt-TL1	Male	10	0.111	0.001	4	0.200	0.000	6	0.052	0.179
	Female	14	0.027		5	0.044		9	0.018	
mt-TQ	Male	8	0.111	0.003	5	0.178	0.086	3	0.000	0.586
	Female	5	0.123		1	0.417		4	0.050	
mt-ND4	Male	25	0.102	0.087	12	0.023	0.425	13	0.174	0.102
	Female	25	0.038		10	0.012		15	0.056	
mt-TS2	Male	17	0.057	0.572	8	0.092	0.783	9	0.025	0.294
	Female	14	0.028		4	0.081		10	0.007	
mt-TE	Male	15	0.139	0.070	7	0.240	0.024	8	0.050	0.834
	Female	18	0.060		7	0.080		11	0.048	
mt-TT	Male	14	0.076	0.005	6	0.166	0.002	8	0.009	0.909
	Female	14	0.034		8	0.044		6	0.019	
D-Loop	Male	21	0.128	0.578	9	0.242	0.118	12	0.043	0.294
	Female	21	0.095		7	0.120		14	0.082	

**Table 7:** Correlation between age and methylation levels of mitochondrial genomic regions that showed significant difference between AD patients and the control subjects.

	Total			Control			AD		
	*N*	r	*p* Value	*N*	r	*p* Value	*N*	r	*p* Value
mt-TF	18	−0.396	0.129	8	0.063	0.894	10	0.620	0.075
mt-RNR1	44	−0.394	0.010	19	0.201	0.424	25	0.467	0.021
mt-TV	24	−0.358	0.102	10	−0.131	0.737	14	0.620	0.024
mt-RNR2	49	−0.199	0.179	22	0.270	0.237	27	0.249	0.221
mt-TL1	24	−0.481	0.023	9	0.745	0.034	15	−0.019	0.948
mt-TQ	13	−0.543	0.084	6	−0.673	0.213	7	0.000	1.000
mt-ND4	50	0.294	0.042	22	0.225	0.326	28	−0.351	0.072
mt-TS2	31	−0.421	0.023	12	0.370	0.263	19	0.197	0.434
mt-TE	33	−0.174	0.350	14	0.223	0.464	19	0.298	0.230
mt-TT	28	−0.556	0.003	14	0.139	0.650	14	0.429	0.143
D-Loop	42	−0.387	0.014	16	0.088	0.755	26	0.237	0.254

## Data Availability

Data described in the article will be made available upon request by researchers for specified scientific purposes via contacting the corresponding authors.

## Supplementary Materials

The following supporting information can be downloaded at: https://www.mdpi.com/article/10.3390/diagnostics13142351/s1, Table S1. Summary of the main clinical of the participants; Table S2. The methylation change levels of D-Loop region between control subjects and AD patients; Table S3. The methylation change levels of mt-ND1 region between control subjects and AD patients.
