# Supplementary material for: Characterization of Mitochondrial DNA Methylation of Alzheimer’s Disease in Plasma Cell-Free DNA
Source: Diagnostics (Basel). 2023 Jul 12;13(14):2351. doi: 10.3390/diagnostics13142351 (PMC10378411; doi:10.3390/diagnostics13142351)
Supplement: Supplementary file 1 [file diagnostics-13-02351-s001.zip › diagnostics-2428905-supplementary.pdf]

Supplementary data

The following are the Supplementary data to this article:

Table S1 Summary of the main clinical of the participants

	Group	Gender	Age	MoCA scores	MMSE scores
Control1	control	male	88	29	
Control2	control	female	78	27	
Control3	control	female	71	30	
Control4	control	female	71	27	
Control5	control	male	77	29	
Control6	control	female	74	27	
Control7	control	female	79	28	
Control8	control	female	79	26	
Control9	control	male	76	29	
Control10	control	male	87	28	
Control11	control	male	79	29	
Control12	control	female	82	29	
Control13	control	female	81	28	
Control14	control	female	76	30	
Control15	control	male	67	27	
Control16	control	male	74	28	
Control17	control	male	72	30	
Control18	control	female	78	28	
Control19	control	female	78	30	
Control20	control	female	82	30	
Control21	control	female	76	30	
Control22	control	male	80	29	
Control23	control	male	77	28	
Control24	control	male	77	29	
Control25	control	male	79	28	
Control26	control	male	71	30	
AD1	case	male	79		3
AD2	case	female	85		10
AD3	case	female	82		13
AD4	case	female	77		8
AD5	case	female	85		6
AD6	case	female	60		6
AD7	case	male	76		0
AD8	case	female	75		7
AD9	case	male	69		0
AD10	case	male	90		12
AD11	case	female	78		0
AD12	case	female	73		6
AD13	case	male	79		2
AD14	case	female	88		10
AD15	case	female	72		11
AD16	case	male	76		12
AD17	case	male	81		9
AD18	case	female	85		9
AD19	case	male	81		10
AD20	case	male	88		8
AD21	case	female	87		15
AD22	case	female	82		7
AD23	case	male	81		5
AD24	case	male	78		9
AD25	case	male	76		6
AD26	case	female	88		13
AD27	case	female	89		13
AD28	case	female	83		8
AD29	case	male	87		10
AD30	case	male	77		0
AD31	case	female	82		6

Table S2 The methylation change levels of D-Loop region between control subjects and AD patients

type	regions	location	methylation change levels	p value
CpG sites	D-loop	chrM:61	−0.146	0.034
	D-loop	chrM:78	−0.102	0.110
	D-loop	chrM:80	−0.090	0.152
	D-loop	chrM:91	−0.085	0.272
	D-loop	chrM:96	−0.188	0.058
	D-loop	chrM:105	−0.161	0.076
	D-loop	chrM:120	−0.151	0.018
	D-loop	chrM:162	−0.179	0.123
	D-loop	chrM:170	−0.204	0.012
	D-loop	chrM:186	−0.124	0.404
	D-loop	chrM:262	−0.116	0.555
	D-loop	chrM:317	−0.086	0.124
	D-loop	chrM:413	−0.140	0.001
	D-loop	chrM:500	−0.117	0.017
	D-loop	chrM:527	−0.156	0.000
	D-loop	chrM:546	−0.106	0.028
	D-loop	chrM:16,084	−0.194	0.037
	D-loop	chrM:16,096	−0.203	0.026
	D-loop	chrM:16,129	−0.100	0.368
	D-loop	chrM:16,330	−0.115	0.087
	D-loop	chrM:16,362	−0.187	0.002
	D-loop	chrM:16,413	−0.248	0.000
	D-loop	chrM:16,429	−0.179	0.001
	D-loop	chrM:16,451	−0.172	0.009
	D-loop	chrM:16,456	−0.192	0.001
	D-loop	chrM:16,497	−0.106	0.001
Methylation regions	D-Loop	chrM:1–100	−0.192	0.039
	D-Loop	chrM:101–200	−0.156	0.036
	D-Loop	chrM:201–300	−0.116	0.555
	D-Loop	chrM:301–400	−0.086	0.124
	D-Loop	chrM:401–500	−0.108	0.002
	D-Loop	chrM:16,101 - 16,200	−0.100	0.368
	D-Loop	chrM:16,301 - 16,400	−0.186	0.001
	D-Loop	chrM:16,401 - 16,500	−0.184	0.000

Table S3 The methylation change levels of mt-ND1 region between control subjects and AD patients

type	regions	location	methylation change levels	p value
CpG sites	MT-ND1	chrM:3352	−0.107	0.070
	MT-ND1	chrM:3376	−0.141	0.028
	MT-ND1	chrM:3380	−0.146	0.050
	MT-ND1	chrM:3407	−0.165	0.121
	MT-ND1	chrM:3421	−0.129	0.042
	MT-ND1	chrM:3436	−0.166	0.031
	MT-ND1	chrM:3454	0.015	0.850
	MT-ND1	chrM:3460	0.040	0.743
	MT-ND1	chrM:3496	0.133	0.256
	MT-ND1	chrM:3526	0.071	0.638
	MT-ND1	chrM:3531	0.033	0.748
	MT-ND1	chrM:3550	0.078	0.521
	MT-ND1	chrM:3643	−0.076	0.082
	MT-ND1	chrM:3688	−0.097	0.001
	MT-ND1	chrM:3697	−0.120	0.000
	MT-ND1	chrM:3700	−0.091	0.022
	MT-ND1	chrM:3707	−0.104	0.003
	MT-ND1	chrM:3890	−0.085	0.145
	MT-ND1	chrM:3901	−0.112	0.018
	MT-ND1	chrM:3910	−0.082	0.061
	MT-ND1	chrM:3922	−0.100	0.031
	MT-ND1	chrM:3946	−0.142	0.003
	MT-ND1	chrM:3952	−0.126	0.018
	MT-ND1	chrM:3955	−0.159	0.003
	MT-ND1	chrM:3967	−0.172	0.016
	MT-ND1	chrM:3985	−0.137	0.174
	MT-ND1	chrM:4127	−0.118	0.151
Methylation regions	MT-ND1	chrM:3401 - 3500	0.052	0.663
	MT-ND1	chrM:3501 - 3600	0.090	0.432
	MT-ND1	chrM:3601 - 3700	−0.057	0.086
	MT-ND1	chrM:3701 - 3800	−0.104	0.003
	MT-ND1	chrM:3801 - 3900	−0.085	0.145
	MT-ND1	chrM:3901 - 4000	−0.103	0.022
	MT-ND1	chrM:4101 - 4200	−0.143	0.068
